# Very Low Volume High-Intensity Interval Exercise Is More Effective in Young Than Old Women

**DOI:** 10.1155/2018/8913187

**Published:** 2018-05-09

**Authors:** Raulas Krusnauskas, Tomas Venckunas, Audrius Snieckus, Nerijus Eimantas, Neringa Baranauskiene, Albertas Skurvydas, Marius Brazaitis, Artemide Liubinskiene, Sigitas Kamandulis

**Affiliations:** ^1^Institute of Sport Science and Innovations, Lithuanian Sports University, Kaunas, Lithuania; ^2^ARS Medica Clinic, Studentų g. 25, LT-50239 Kaunas, Lithuania

## Abstract

We investigated the acute neuromuscular and stress responses to three different high-intensity interval training sessions in young (age 19.5 ± 1.3 years) and older (age 65.7 ± 2.8 years) women. Cycling exercise comprised either 6 × 5 s or 3 × 30 s all-out, or 3 × 60 s submaximal, efforts each performed 5 weeks apart in randomized order. Peak and average power was higher in young than in older women and was largest during the 6 × 5 s strategy in both groups (*p* < 0.05). The decrease in the ratio of torques evoked by 20 and 100 Hz electrical stimulation, representing low-frequency fatigue, was more evident after the 3 × 30 and 3 × 60 s than the 6 × 5 s bout in both groups and was larger in young than in older women (*p* < 0.05). Both groups preferred 6 × 5 s cycling for further training. In conclusion, in young women, very low volume (6 × 5 s) all-out exercise induces significant physiological stress and seems to be an effective means of training. For older women, longer exercise sessions (3 × 60 s) are more stressful than shorter ones but are still tolerable psychologically.

## 1. Introduction

Reduced physical activity in older adults is associated with increased risk of disease and mortality [1, 2], and it is well established that exercise training reduces the risk of disease and disability [3]. High-intensity interval training (HIIT) has been proposed as an effective and time efficient method for increasing aerobic capacity and metabolic health in young and older adults [3–8]. HIIT sessions are typically short (15–300 s), intense (70–100% of maximum) bursts of activity interspaced with 1–5 min of rest [9]. This training differs from traditional endurance training that involves prolonged and continuous exercise supplied mainly by aerobic energy system [10, 11].

There is little agreement on the extent to which the benefits of HIIT can be attributed to the exercise intensity alone among the other load variables [12, 13]. Training at both moderate to high (70–80%) and near-maximal (>90%) intensity can significantly improve health parameters [13]. Place et al. [7] have shown that repetitive maximal intensity (all-out) 30 s cycling bouts induce a long-lasting increase in resting intracellular sarcoplasmic calcium concentration ([Ca^2+^]i), which triggers the mechanism responsible for adaptations. These findings favor “higher-the-better” strategy when considering exercise intensity. All-out efforts of HIIT currently prevails in increasing endurance of various age and physical condition groups [4, 14, 15].

Despite the well-documented HIIT benefits, it is important to consider that very intense exercise can cause significant build-up of metabolites that can induce unpleasant sensations [6]. Practical observations during the laboratory experiments showed that as little as one 30 s all-out cycling bout may induce dizziness or collapse, even in well-trained athletes. These side effects could diminish motivation and preclude systematic and long-term implementation of all-out HIIT in general population. Moreover, because of side effects issues, it is doubtful whether prescription of maximal intensity HIIT to some specific populations such as older people is safe and effective. At the same time, moderate to high-intensity training has been shown to provide beneficial effects even for patients with a chronic disease [16].

These challenges have encouraged further searches for HIIT modalities that are enjoyable and time efficient and yet still confer substantial benefits for health and exercise capacity. One specific issue relates to determination of the minimal effective HIIT volume. It has been shown that as few as 12 bouts of 5 s all-out cycling can induce a long-lasting decrease in the plasma concentration of brain-derived neurotrophic factor in young men [17], which may be beneficial to brain health. In the present study, we aimed to analyze the acute effects of three different HIIT sessions of submaximal to all-out intensity on neuromuscular and stress responses in young and older women.

We hypothesized that very low volume HIIT session (6 bouts of 5 s) would be tolerated comfortably and provide enough stimulus to surpass threshold needed for physiological response. Bearing in mind age related differences in physical and cognitive abilities [18], we also hypothesized that young and old women would differ in their physiological response to HIIT.

## 2. Materials and Methods

### 2.1. Participants

The characteristics of the participants are shown in Table 1. A medical doctor checked their health and excluded any volunteer from the study if any cardiovascular complaint, sign of metabolic disease, or chronic joint pain was noted in the examination. In order to better characterize groups, participants completed short version of the International Physical Activity Questionnaire [19, 20] and also the World Health Organization quality of life questionnaire (WHOQOL-100) [21] before the study. Recruited participants were involved in different recreational activities (e.g., jogging, dancing, and swimming) 1 to 2 times per week and were encouraged to maintain their exercise routine during the study. They were also instructed to abstain from any exercise 2 days before each experimental intervention. All procedures were approved by the Kaunas Region Biomedical Research Ethics Committee. The participants read and voluntarily signed an informed consent form consistent with the principles outlined in the Declaration of Helsinki.

### 2.2. Familiarization and Warm-Up

A familiarization session was performed 7 days before the first cycling exercise. The participant was seated in the dynamometer chair (System 3; Biodex Medical Systems, Shirley, NY, USA) and was asked to perform a few attempts of maximal knee extension. Tolerance to electrical stimulation was assessed during the same visit. To familiarize each participant with the stationary cycling ergometer (Monark 824E; Monark, Vansbro, Sweden), subjects performed several 10 s cycling bouts with a brake weight of 3.75% of body weight. Only those participants who showed good compliance with the procedures were recruited for the study.

Warm-up was performed, and the baseline measures of strength were obtained. These measurements were repeated 1 h and 24 h after exercise. The warm-up comprised 7-8 min of stationary cycling with the power set so that the number of watts equaled the participant's body weight in kilograms. After the warm-up, the participant performed three trials of knee extension at 50, 70, and 90% of maximal effort before measurement of maximal force, as described below.

### 2.3. Assessment of VO_2_ Peak

The VO_2_ peak test was performed within 1 week before the first cycling session. Gas analysis was performed using a portable breath-by-breath analyzer (Oxygen Mobile; Jaeger/VIASYS Healthcare, Hoechberg, Germany). The test began with 3 min of cycling at 20 W, after which the intensity increased steadily by 3 W every 10 s with a constant pedaling rate of about 60 rpm until volitional fatigue. Heart rate (HR) was monitored throughout the test (S-625X; Polar Electro, Kempele, Finland).

### 2.4. Cycling Exercise

Three different types of cycling exercise were performed on a mechanically braked cycle ergometer (Monark 824E) by all participants in random order, with each test interspaced by a 5-week rest period to completely abolish the effects of previous intervention. The 6 × 5 s very low volume exercise comprised six repetitions of 5 s of all-out cycling with the brake weight equal to 7.5% of the participant's body weight with a rest period of 90 s between each repetition. The 3 × 30 s exercise comprised three bouts of 30 s of all-out cycling with a 7.5% brake weight and a 4 min rest period between each repetition. The 3 × 60 s exercise comprised three bouts of 60 s of submaximal cycling with a 3.75% brake weight and 4 min rest period between each repetition. During the rest periods, the participant cycled against minimal resistance at a comfortable rate. Peak and average power were used in the analyses, and HR was measured during all cycling exercises.

### 2.5. Maximal Voluntary Contraction (MVC) and Electrically Evoked Torques

MVC peak torque was measured for the knee extensor muscles on the dominant leg using an isokinetic dynamometer (System 3; Biodex Medical Systems). The participant sat in the dynamometer chair with the knee joint positioned at a 60° angle (0° = full knee extension). MVC was reached and maintained for ~2 s before relaxation and was measured twice; the larger value was used in the analysis. Direct muscle stimulation was applied using two carbonized rubber surface electrodes covered with a layer of electrode gel (Medigel; Modi'in, Israel). Electrodes were positioned over both the proximal and distal ends of the quadriceps femoris. A standard electrical stimulator (MG 440; Medicor, Budapest, Hungary) was used to deliver supramaximal 0.5 ms square-wave pulses for 1 s trains of stimuli at frequencies of 20 (P20) and 100 (P100) Hz. Changes in the P20/P100 ratio after exercise were used to evaluate low-frequency fatigue (LFF) [22, 23].

### 2.6. Perceived Exertion and HIIT Type Preference

The rating of perceived exertion (RPE) Borg et al. [24] was reported immediately after exercise. After completion of each of the three HIIT sessions, each participant was asked the following questions. (1) Which of the three exercise modalities would you prefer for further training? (2) Which of the nonpreferred two exercise modalities would you choose for further training? Finally, the participant was asked to indicate the reasons for her exercise preference. Similar methodology has been used in previous studies [15, 25].

### 2.7. Blood Analyses

Venous blood samples (5 ml) were collected at the baseline and then 5 min, 1 h, and 24 h after exercise. The blood was allowed to clot at room temperature, and the serum was separated by centrifugation for 15 min at 1,200*g*. The concentrations of insulin-like growth factor 1 (IGF-1) and tumor necrosis factor *α* (TNF-*α*) were measured using an enzyme-linked immunoassay system (LDN; Immunoassays and Services, Nordhorn, Germany, and DIAsource ImmunoAssays; Louvain-la-Neuve, Belgium, respectively). Fingertip blood lactate concentration was measured at the baseline, 5 min, and 1 h after cycling exercise using a portable lactate analyzer (Pro™ LT-1730; Arkray Inc., Kyoto, Japan).

### 2.8. Statistical Analyses

Data are presented as mean ± standard deviation (SD). The study was powered using low-frequency (20 Hz) stimulation evoked knee extension torque as the primary outcome variable based on LFF as indirectly index of disturbances in excitation–contraction coupling and ion handling [26]. For a One-Tailed hypothesis with *α* = 0.05 and statistical power level 0.80, the minimum number of participants required to detect a change was nine in each group according to changes in a previous study [23].

Before the analyses, the Kolmogorov–Smirnov test was used to check the normality of data distribution. For normally distributed data, three-way (group, time, and session type) analysis of variance (ANOVA) with repeated measures was used to compare differences in outcome measures (MVC, P100, P20, P20/P100 ratio, and concentrations of lactate, IGF-1, and TNF-*α*). If a significant interaction effect was found, further analyses were performed using two-way (time and session type) ANOVA with repeated measures separately for each age group. Post hoc testing was performed using Tukey's correction for multiple comparisons. Two-way analysis of variance was also used to identify differences in peak and average force and HR within groups during the different cycling sessions. A *t* test was used to identify differences in physical characteristics between the age groups. The IPAQ and WHOQOL-100 scores were compared between the young and older groups using the nonparametric Mann–Whitney *U* test. A chi-square test was used to compare the distribution of preferences for the different session types. Significance was set at *p* < 0.05.

## 3. Results

### 3.1. Baseline Measurements

Body weight and body mass index were significantly greater in the older women, but lean body mass did not differ between groups (*p* > 0.05, Table 1). VO_2_ peak and peak power attained during the progressive cycling were significantly higher in the young women. The amount of self-reported PA did not differ significantly between age groups, both of which had an average > 3,000 METs per week. Young women had significantly better QOL in all domains except for general health and spirituality (Table 2).

### 3.2. Cycling Exercise

**Figure 1:**
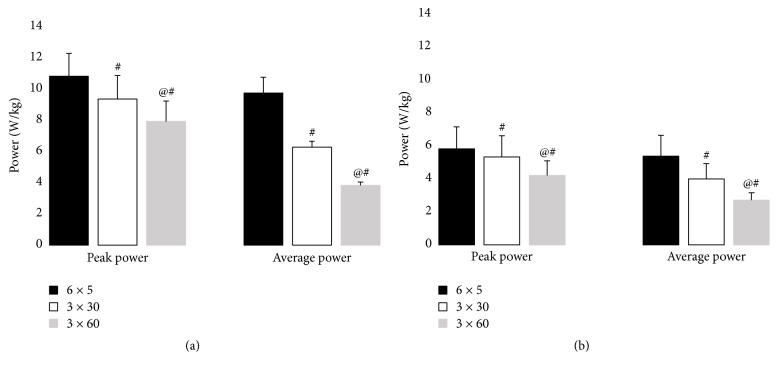
Peak and average power during cycling exercise in the (a) young group and (b) older group, ^#^*p* < 0.05 compared to 6 × 5 s cycling exercise; ^@^*p* < 0.05 compared to 3 × 30 s cycling exercise.

Two-way ANOVA showed a strong interaction effect of group and session type on peak and average power (*p* < 0.05, Figure 1). Peak and average power values were consistently higher in young than in older women and were highest for the 6 × 5 s sessions and lowest for the 3 × 60 s sessions for both groups (*p* < 0.05). For the young women, the average exercise intensity during the bout relative to peak power was 95.6% ± 1.6%, 82.4% ± 8.1%, and 69.5% ± 5.5% during the 6 × 5 s, 3 × 30 s, and 6 × 30 s cycling, respectively. The respective values for older women were 92.8% ± 5.0%, 85.0% ± 8.0%, and 67.4% ± 3.7% (*p* < 0.01 compared between session types; *p* > 0.05 compared between groups). In young women, the average work done during 6 × 5 s, 3 × 30 s, and 6 × 30 s cycling sessions was 293.3 ± 30.5 J/kg, 566.6 ± 34.5 J/kg, and 691.3 ± 38.4 J/kg, respectively. The respective values for older women were 163.2 ± 38.2 J/kg, 364.3 ± 83.2 J/kg, and 493.3 ± 81.1 J/kg (*p* < 0.01 compared between session types; *p* > 0.05 compared between groups).

The highest peak HR was achieved during the 3 × 60 s session (184.5 ± 9.0 bpm and 157.8 ± 21.5 bpm for the young and older groups, respectively; *p* < 0.01). The lowest peak HR was achieved during the 6 × 5 s session (169.2 ± 7.3 bpm and 125.1 ± 14.1 bpm for the young and older groups, respectively; *p* < 0.001). In the young group, the HR percentages relative to peak HR were 87.7% ± 3.8%, 93.8% ± 3.2%, and 95.6% ± 4.1% during the 6 × 5 s, 3 × 30 s, and 6 × 30 s sessions. In the older group, the respective values were 79.3% ± 5.0%, 92.5% ± 5.9%, and 98.4% ± 5.9% (*p* < 0.05 for the 6 × 5 s versus the other sessions; *p* < 0.05 between the young and older groups for the 6 × 5 s session).

**Figure 2:**
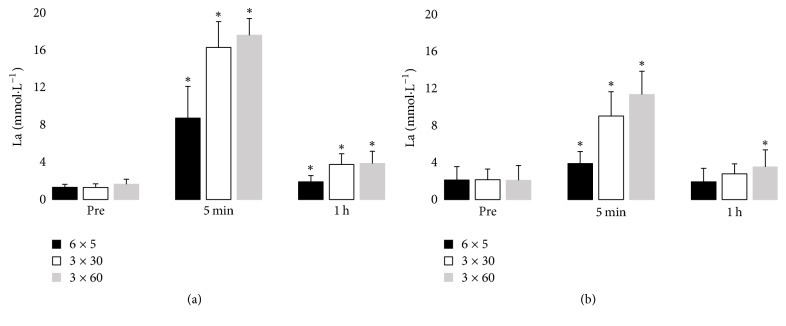
Blood lactate levels before and after HIIT cycling exercise in the (a) young group and (b) older group, ^*∗*^*p* < 0.05 compared to preexercise (Pre) value.

Blood lactate concentration showed a significant interaction between time and HIIT type, in both age groups (*p* < 0.05, Figure 2). In all three exercise sessions, the lactate concentration was significantly higher in the young group than in the older group (*p* < 0.05). Lactate concentration was highest 5 min after the 3 × 30 s and 6 × 30 s cycling sessions in both age groups. In the young group, blood lactate concentration remained above the baseline level 1 h after all three HIIT session types. In the older group, blood lactate concentration remained above the baseline level only after the 3 × 60 s session.

### 3.3. Perceptual Measures

Ratings of perceived exertion were 12.3 ± 2.5, 16.9 ± 1.5 and 16.4 ± 1.6 points for young and 12.8 ± 1.3, 15.8 ± 1.5 and 15.9 ± 1.8 points for older women during 6 × 5 s, 3 × 30 s, and 6 × 30 s cycling, respectively (*p* < 0.05 for 6 × 5 s versus the other two types of HIIT, *p* > 0.05 compared between young and older groups). Women of both age groups ranked the 6 × 5 s cycling as the most preferred for further training (90% of all participants, *p* < 0.05). The 3 × 30 s and 6 × 30 s sessions were ranked similarly by both groups (about 50% of all participants, *p* > 0.05), indicating that they had no preference for the second-most preferred session type. Low stress and high enjoyment were reported most frequently as the main reasons for selecting the desired exercise type. Interestingly, the same reason, too little stress, was given by 10% of all participants for not preferring the 6 × 5 s strategy.

### 3.4. MVC

**Figure 3:**
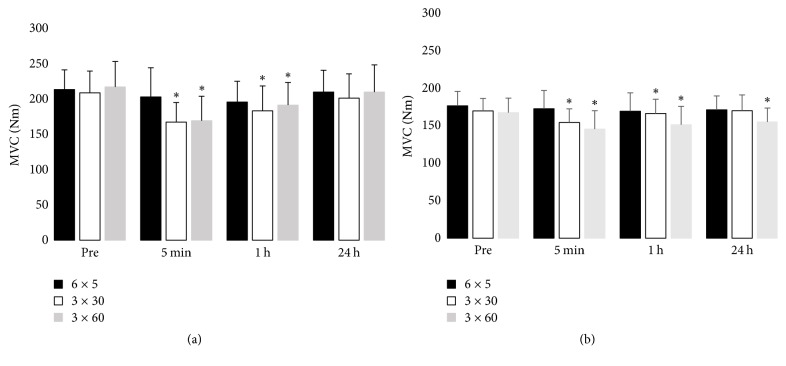
MVC peak torque before and after cycling exercise in the (a) young group and (b) older group, ^*∗*^*p* < 0.05 compared to preexercise (Pre) value.

There was a significant interaction between time and session type for MVC in both age groups (*p* < 0.05, Figure 3). In young women, MVC decreased 5 min and 1 h after 3 × 30 s and 3 × 60 s cycling but recovered completely by 24 h after exercise. Similarly, in older women, MVC decreased significantly 5 min and 1 h after 3 × 30 s and 3 × 60 s cycling but remained low at 24 h after the 3 × 60 s cycling session.

### 3.5. Electrically Induced Muscle Torque and LFF

**Figure 4:**
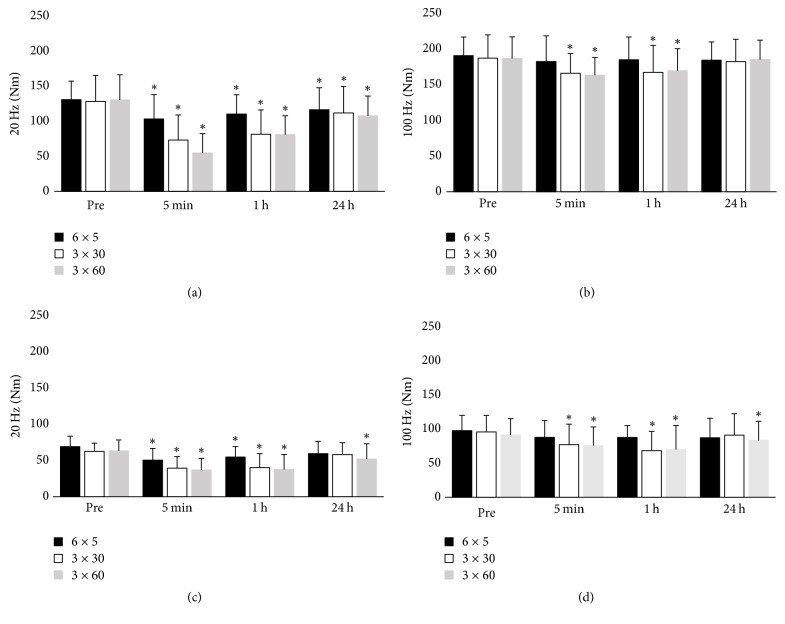
Electrically induced muscle performance at low (P20) and high (P100) frequency before and after cycling exercise in the (a) and (b) young group and (c) and (d) older group, ^*∗*^*p* < 0.05 compared to preexercise (Pre) value.

**Figure 5:**
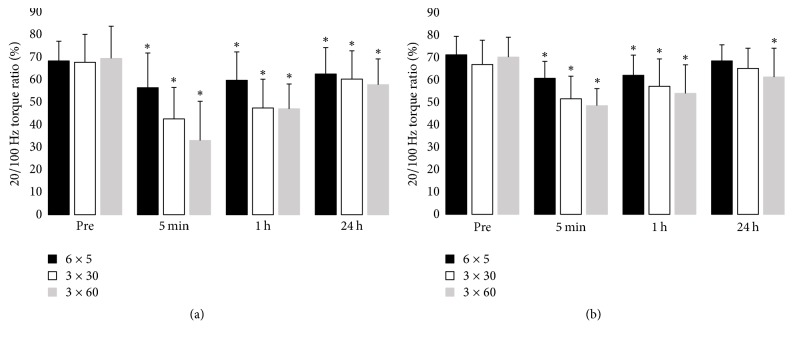
P20/P100 ratio, a measure of LFF, before and after cycling exercise in the (a) young and (b) older group. ^*∗*^*p* < 0.05 compared to preexercise (Pre) value.

There was significant interaction of session type and time for P20 and P100 (*p* < 0.05, Figure 4). P100 was unchanged after the 6 × 5 s session in both groups but had decreased after rest sessions and remained low at 24 hours after the 3 × 60 s session for older women only (*p* < 0.05). P20 decreased significantly after all types of cycling sessions in both age groups and had not recovered within 24 h in the young group and after the 3 × 60 s session in the older group. A decline in the P20/P100 ratio, which represents LFF, was more evident after the 3 × 30 s and 3 × 60 s sessions than after the 6 × 5 s session in both groups and was larger in the young group (*p* < 0.05, Figure 5). In addition, the decline in LFF remained significant in the young group at 24 h after all types of HIIT, whereas it had recovered fully by 24 h after the 6 × 5 s and 3 × 30 s sessions in the older group.

### 3.6. Blood Analysis

TNF-*α* concentration tended to be higher in older women (9.8 ± 5.1 ng/ml and 14.4 ± 7.8 ng/ml for the young and older groups, respectively; *p* = 0.07), but there was no interaction between time and HIIT session type for TNF-*α* in either of the groups (*p* > 0.05). IGF-1 concentration was higher in young women (246.3 ± 118.1 ng/ml and 141.4 ± 41.2 ng/ml for the young and older groups, respectively; *p* < 0.05), but the changes after exercise were minor in both age groups irrespective of the HIIT session type (*p* > 0.05).

## 4. Discussion

The major finding of this study was that extremely low volume, repeated high-intensity exercise caused a long-lasting (>24 h) decrease in LFF in young women. Older women tended to respond more to, and thus benefit more from, the longer duration, high-intensity exercise bouts, which suggests that a higher total volume of exercise is required to stimulate adaptations in older women. Perceived enjoyment of the HIIT sessions was similar for both groups, which preferred the lower volume session with shorter bouts. These observations partly confirm the hypothesis that the lowest volume strategy is best tolerated but still effective, even though it was less so in older women.

### 4.1. LFF and Metabolic Stress in Very Low Volume HIIT

Enhanced ion handling during the initial phases of adaptation has been proposed as the main mechanism for improved endurance after exercise training [27]. In the present study, we assessed LFF, which indirectly indicates disturbances in excitation–contraction coupling in muscles [26]. Skeletal muscle fibers are activated by Ca^2+^ release from internal stores (i.e., the sarcoplasmic reticulum) via the ryanodine receptor 1 protein complex. Force depression occurs because of reduced Ca^2+^ release, which leads to decreased free myoplasmic [Ca^2+^]i and/or decreased myofibrillar Ca^2+^ sensitivity [28]. This would result in a larger force depression at low than at high stimulation frequencies because of the sigmoidal shape of the force–[Ca^2+^]i relationship. Changes in Ca^2+^ kinetics occur because acute metabolic stress in muscle fibers is accompanied by an increase in the release of reactive oxygen/nitrogen species [29–33] which can stimulate mitochondrial biogenesis and eventually increase endurance [32]. Here, we observed a disturbance in excitation–contraction coupling that lasted for at least 1 h in older women and for at least 24 h in young women after only six 5 s sprints (i.e., a total of 30 s of all-out exercise). This is an encouraging finding and suggests that extremely low volume but metabolically demanding exercise may be able to activate the cellular signaling pathways and thereby improve muscle endurance and metabolic health.

The differences in LFF between the young and older women in response to these types of very low volume, high-intensity exercise may be explained by different levels of metabolic stress. Older women were unable to attain high power production during the 5 s bouts to the same level as did young women, and this was reflected in the lower lactate concentration and lower HR percentage relative to maximal HR in the older women. The lower exercise-induced stress may preclude full-scale adaptations, which may require a longer total duration of HIIT, particularly in older women.

We note that MVC, P100, and IGF-1 and TNF-*α* concentrations did not change in either group after the 6 × 5 s all-out cycling; this lack of response suggests that impaired Ca^2+^ handling may be the main mechanism responsible for the effects of extremely low volume, high-intensity training. Such mechanism could be activated only if the metabolic demands are large enough to induce long-lasting LFF.

### 4.2. Exercise Volume and Enjoyability

The findings of our study confirm the strategy of “more is better.” High-intensity cycling for a longer duration induced greater metabolic stress in both young and older women. It was not surprising that the longer exercise was rated as less enjoyable and less preferable for long-term training. This is consistent with previous studies that have compared different exercise volume strategies [14, 15, 25]. It is notable that rather minor differences in physiological and psychological stress were found between the 3 × 30 s and 3 × 60 s cycling sessions despite differences in exercise intensity and volume. The main differences in the response between these two types of training sessions were longer LFF and MVC depression in older subjects, which confirm the importance of exercise volume for aged women. Despite similar general daily activity patterns in the two groups, it is possible that older women were doing less vigorous activity and exhibited some degree of general frailty because of their age; if so, this may have made it difficult to perform the intense cycling exercise, especially the sessions involving 5 s all-out intervals. Although speculative, this may indicate that an impaired ability to exercise maximally necessitates prescription of some longer intervals and a greater total duration of intense exercise for older persons to achieve the same cumulative stress and health benefits as in younger exercisers. Interestingly, older women rated the psychological stress of both longer (3 × 30 s and 3 × 60 s) strategies similarly, which suggests that both types of HIIT sessions were perceived as enjoyable despite the 3 × 60 s training session being more stressful. Therefore, longer bouts could be applied in training without endangering the safety or tolerability in older women.

### 4.3. Blood Cytokines and Hormones Responses to HIIT

Cytokines are important mediators of various aspects of health and disease, including glucose and lipid metabolism, insulin sensitivity, and skeletal muscle maintenance. We found that the concentration of the inflammatory marker TNF-*α* did not differ significantly between young and older women at baseline and did not change consistently in response to any of the low volume HIIT sessions. IGF-1 concentration was reduced in older women; given the specifics of IGF-1-triggered signaling pathways, this may be associated with impaired muscle maintenance and muscle function in the older women. The changes in IGF-1 concentration in response to the HIIT sessions were minor and not significant in any age group, and it seems unlikely that this cytokine is involved in the age related differences (if they exist) in adaptations to low volume HIIT, at least in women.

### 4.4. Limitations and Future Perspectives

One limitation of the study is that maximal power was recorded in 5 s intervals, and it is possible that the older women did not achieve maximal power in such a short period. Therefore, the intensity relative to maximal power might have been overestimated in the old women group. In addition, the interventions were not matched by performed work that made it impossible to estimate the effect of exercise intensity per se. One more limitation was the large variability in the blood cytokine responses making it difficult to detect significant changes and conclude how different sprint training protocols affect these markers. Finally, we did not investigate brain mechanisms involved in the present findings while future studies should evaluate brain health markers (BDNF-alpha, kyrunenin) response to very low volume HIIT sessions. Furthermore, future research should extend the observations of the current study to male gender and different populations such as athletes, patients, and other groups.

## 5. Conclusions

Very low volume, high-intensity exercise (6 × 5 s) induces significant physiological stress and seems to be an effective means of training, especially in young women. For older women, more voluminous exercise sessions (3 × 60 s) seem to be more stressful than shorter ones but are still tolerable psychologically. Short maximal intensity exercise bursts can be recommended for both young and older women but practitioners who plan such training programs should consider age effect on the physiological and psychological responses.

## Figures and Tables

**Table 1:** Subjects' characteristics and fitness results in young and older women.

	Young *N* = 10	Old *N* = 9	*p* value (unpaired *t*-test)
Age (Y)	19.5 ± 1.3	65.7 ± 2.8	<0.001
Height (cm)	169 ± 4.8	164.4 ± 6.3	0.094
Body mass (kg)	62.1 ± 8.5	75.8 ± 15.02	0.024
Body mass index (kg/m^2^)	21.6 ± 2.3	27.9 ± 5.3	0.003
Fat free mass (%)	47.7 ± 3.8	44.5 ± 4.9	0.138
V0_2_ Peak (ml/kg/min)	32.9 ± 4.9	20.4 ± 6.1	<0.001
Peak heart rate (b/min)	192.3 ± 8.1	150.2 ± 20.4	0.002
Peak power (W)	221.7 ± 31.1	152.6 ± 22.6	<0.001

Values given are mean ± standard error of the mean.

**Table 2:** The comparison of WHOQOL-100 scores between young and older women.

The WHOQOL-100 domains	Young women (*n* = 10)	Older women (*n* = 9)	*p* value (unpaired *t*-test)
Overall QOL	85.0 ± 11.5	73.6 ± 12.0	0.059
Physical	77.3 ± 8.5	59.7 ± 14.1	0.011
Psychological	81.5 ± 9.1	65.1 ± 4.5	<0.001
Level of independence	92.0 ± 4.1	70.0 ± 16.9	0.002
Social relationships	88.3 ± 11.3	63.2 ± 9.4	0.001
Environment	79.4 ± 3.7	71.2 ± 7.6	0.019
Spirituality	79.4 ± 18.6	73.6 ± 14.2	0.450

Values given are mean ± standard error of the mean. WHOQOL: World Health Organization Quality of life as individuals.
